# Multimodal Characterization of Radiation-Induced Optic Neuropathy after Proton Radiotherapy

**DOI:** 10.1016/j.xops.2026.101359

**Published:** 2026-08-10

**Authors:** Juliette Thariat, Thao-Nguyen Pham, Thibaud Mathis, Dorothee Lebhertz, Jean-Claude Quintyn

**Affiliations:** 1 Department of Radiation Oncology, Centre François Baclesse, Caen, France; 2 Laboratoire de physique corpusculaire UMR6534 IN2P3/ENSICAEN, Université de Caen- Normandie, Caen, France; 3 Service d’Ophtalmologie, Hôpital de La Croix-Rousse, Hospices Civils de Lyon, Lyon, France; 4 Department of Ophthalmology, University Hospital, Caen, France

**Keywords:** Optic neuropathy, Radiotherapy, Proton therapy, Visual fields, Optical coherence tomography

## Abstract

**Purpose:**

To evaluate the performance of multimodal ophthalmologic assessment compared with current visual acuity (VA)–based on Common Terminology Criteria for Adverse Events classification evaluation of radiation-induced optic neuropathy (RION) severity grade and to describe the relative prominence of functional, electrophysiological, and structural changes across postradiotherapy time windows.

**Design:**

Prospective cohort study.

**Subjects:**

Patients treated with proton radiotherapy for brain, skull base, or head-and-neck tumors between 2018 and 2023 who underwent standardized baseline and longitudinal ophthalmologic evaluations. Eyes with preexisting optic neuropathy were excluded. Of 238 patients, 89 met inclusion criteria (full assessment, no baseline deficit); 39 developed clinically diagnosed RION during follow-up.

**Methods:**

Participants underwent repeated assessments including best-corrected VA (ETDRS), visual field (VF) perimetry (mean deviation [MD] and pattern standard deviation [PSD]), spectral-domain OCT retinal nerve fiber layer (RNFL) thickness, and pattern-reversal visual evoked potentials (VEPs) (P100 latency and amplitude). The median follow-up after proton radiotherapy was 3.83 (2.94–4.75) years. Single-variable and multivariable decision tree models were constructed to identify RION. Random forest models quantified feature importance and temporal dynamics across predefined postradiotherapy intervals (0–0.5, 0.5–1, 1–2, and 2–5 years).

**Main Outcome Measures:**

Model performance (accuracy and F1 score) for identification of clinically diagnosed RION and relative importance of ophthalmologic parameters over time.

**Results:**

Of 39 patients with RION and 50 without, VF MD and PSD demonstrated the strongest individual performance for RION identification (accuracy 75%–79%; F1 score 0.78–0.81), outperforming VA, RNFL thickness, and VEP measures. The multimodal model combining all assessments achieved higher apparent-sample performance (accuracy 81.7% [95% confidence interval (CI), 73.9%–88.1%]; F1 score 0.83 [95% CI, 0.75–0.89]); cross-validated performance was more modest (mean area under the curve 0.64). Random forest analyses identified VF MD as the most influential parameter, followed by VEP measures and longitudinal RNFL change. Within the first year after radiotherapy, functional (VF) and electrophysiological (VEP) abnormalities were more prominent, whereas structural RNFL thinning became more evident between 1 and 2 years. Cross-sectional differences in VA were limited.

**Conclusions:**

In patients with clinically diagnosed RION after proton radiotherapy, functional alterations predominated during the first year after treatment, whereas structural RNFL thinning became evident in the second year. Visual field–based assessment demonstrated greater sensitivity than VA-based grading alone; the cross-validated multimodal model achieved modest performance and did not clearly outperform VF testing alone. These findings should be considered hypothesis-generating. Prospective validation in larger cohorts is warranted.

**Financial Disclosure(s):**

The author has no/the authors have no proprietary or commercial interest in any materials discussed in this article.

Radiation-induced optic neuropathy (RION) is a rare but devastating late complication of radiotherapy involving the optic nerve and chiasm. It typically presents months to years after irradiation, often with abrupt visual loss that is largely irreversible once clinically apparent.^1^^,^^2^ The underlying pathophysiology is complex and multicompartmental, involving radiation-induced microvascular injury, chronic hypoperfusion, demyelination, axonal degeneration, and progressive loss of retinal ganglion cells. These mechanisms are thought to be driven by oxidative stress and persistent molecular and cellular reprogramming following irradiation, explaining both the delayed onset and limited reversibility of the disease.^3^

Despite advances in radiotherapy techniques and dose constraints, RION continues to represent a significant clinical burden.^4^ Its incidence may be underestimated, in part because diagnosis is not standardized and often relies on nonspecific clinical criteria. Current grading systems, such as the Common Terminology Criteria for Adverse Events (CTCAE), primarily emphasize changes in visual acuity (VA).^5^ However, VA predominantly reflects macular function and can be affected by radiation-induced lens or retinal changes, making it an insensitive and nonspecific marker of early optic nerve dysfunction.^6^, ^7^, ^8^

Growing evidence suggests that paraclinical ophthalmologic assessment can detect subclinical optic nerve injury before VA declines. Visual field (VF) testing can reveal early spatially localized functional deficits,^9^^,^^10^ visual evoked potentials (VEPs) can identify conduction abnormalities along the visual pathway,^11^^,^^12^ and OCT enables quantification of retinal nerve fiber layer (RNFL) thinning as a marker of axonal loss.^13^ Importantly, these modalities capture complementary aspects of optic nerve structure and function and may evolve at different stages of the disease process.^14^^,^^15^ However, the relative clinical value of each modality, their relative prominence across postradiotherapy time windows, and their combined ability to identify RION remain incompletely defined. Most prior studies have focused on single modalities, cross-sectional analyses, or dosimetric predictors, limiting their applicability for longitudinal monitoring.

The present prospective study aims to characterize RION using a comprehensive multimodal ophthalmological approach. Specifically, we sought to determine which functional, electrophysiological, and structural parameters are most strongly associated with clinically diagnosed RION. We further evaluated whether combining modalities improves identification compared with single tests and described how the relative relevance of these parameters evolves over time following radiotherapy.

## Methods

### Study Design and Population

A prospective cohort of patients treated with radiotherapy with protons for CNS, skull base, or head-and-neck tumors was established in 2018 under institutional review board approval (Centre François Baclesse, Caen INT-24-016 and University Hospital, Caen CLERS-5496) and in agreement with the Declaration of Helsinki and the General Data Protection Regulation. All patients provided informed consent. All patients underwent standardized ophthalmologic (VA, VF, and OCT) and electrophysiological (VEP) assessments before radiotherapy and at regular intervals thereafter. Eligible patients had a baseline (preradiotherapy) ophthalmologic evaluation and at least one follow-up assessment within 5 years after treatment. Eyes with pre-existing optic neuropathy due to tumor compression, surgical injury, or ocular disease were excluded. To minimize confounding from advanced baseline impairment, analyses were restricted to eyes with a VA score ≥70 ETDRS letters (the CTCAE threshold for optic neuropathy) and a VF mean deviation (MD) better than –6 dB at baseline.

### Ophthalmologic Assessments

Assessments included best-corrected VA (BCVA) measured using the ETDRS scale, MD and pattern standard deviation [PSD] obtained on 30° VF perimetry (Metrovision), spectral-domain OCT-derived mean RNFL thickness (HRA Spectralis, Heidelberg Engineering), and pattern-reversal VEPs characterized by P100 latency and amplitude (Metrovision). Baseline values were defined as the last assessment prior to radiotherapy, and longitudinal changes were calculated relative to baseline. Quality control was performed. OCT scans were accepted based on image quality (Q score) and ophthalmologist verification of retinal layer segmentation. Visual field reliability was assessed using fixation losses. Visual evoked potential recordings were visually reviewed for waveform quality, and repeat acquisitions were performed only when recordings appeared unreliable or aberrant to confirm reproducibility.

### Clinical Definition of RION

Radiation-induced optic neuropathy status was determined as determined by the treating clinicians based on structured clinical annotations during follow-up, with knowledge of ophthalmic assessments. Eyes were classified as having RION when visual impairment was attributed to radiation-induced optic nerve toxicity and not to tumor progression, recurrence, or compressive effects, as determined by the treating clinicians. It should be noted that the treating clinicians had access to all ophthalmic assessments, including VA, VF, OCT, and VEP, as part of routine prospective monitoring, and these same measures were subsequently used as model predictors. This overlap introduces potential verification bias, which readers should consider when interpreting model performance estimates.

### Statistical and Modeling Approach

To evaluate the ability of individual ophthalmologic parameters to identify RION, we fitted single-variable decision tree models using each assessment independently. A multivariable decision tree model incorporating all modalities was then constructed to evaluate the added value of multimodal assessment. Model performance was quantified using accuracy and F1 score, with 95% confidence intervals (CIs) estimated via bootstrap resampling. For all analyses, the unit of analysis was the patient. At each time point, patient-level values were derived from the worse-performing eye (lower VA, more negative VF MD, thinner RNFL, more prolonged VEP P100 latency, or lower VEP P100 amplitude). For patients with unilateral RION, the affected eye was used; for bilateral RION, the worse eye was selected. The same eye was used consistently across all time points for each individual patient. All analyses used a complete-case approach; patients lacking any assessment at a given time window were excluded from that window's analysis, and no imputation was performed.

Random forest models were subsequently used to explore the relative clinical contribution of each parameter and their changes over time. Feature importance was quantified using the mean decrease in Gini impurity across multiple runs with different random seeds. Partial dependence plots (PDPs) were generated to visualize the marginal effect of each variable on the probability of RION classification.

To assess temporal dynamics, analyses were repeated within predefined postradiotherapy intervals (0–0.5, 0.5–1, 1–2, and 2–5 years). Finally, directed correlation networks were constructed to examine temporal relationships between functional, electrophysiological, and structural parameters across successive follow-up periods. All analyses and visualizations were conducted via R (version 4.3.1).

## Results

### Population Characteristics

**Figure 1:**
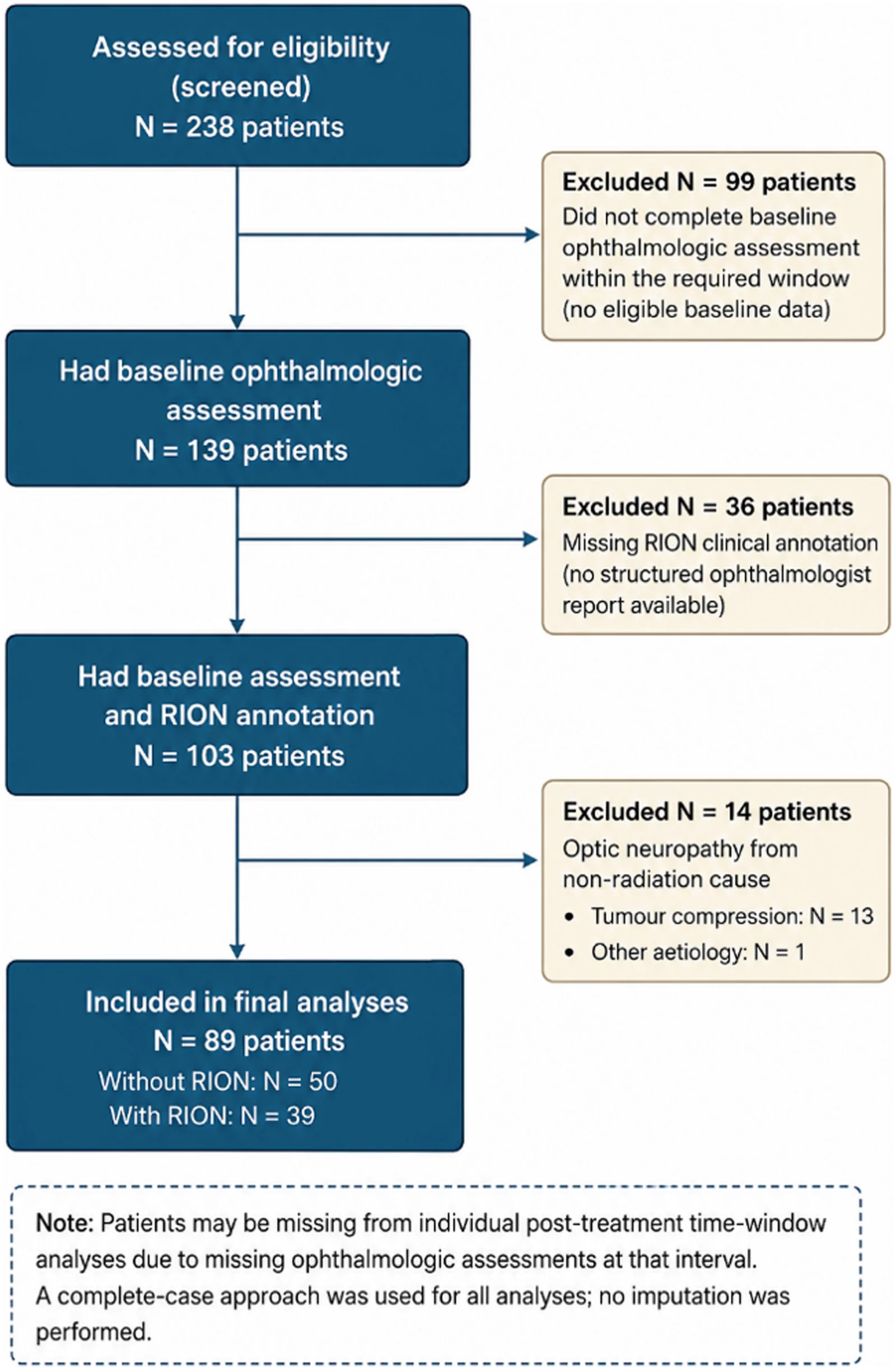
Flowchart of patient selection. RION = radiation-induced optic neuropathy.

Among the 238 screened patients, 139 met baseline ophthalmologic inclusion criteria (Fig 1: Flowchart). Clinical annotation of RION status was available for 103 patients, of whom 50 had no optic neuropathy, 39 developed RION, and the remaining 14 were excluded due to optic neuropathy secondary to tumor compression (n = 13) and optic neuropathy from another cause (n = 1). Among the 89 participants finally included (50 without RION, 39 with RION), baseline VA, RNFL thickness, and VEP parameters were comparable before radiotherapy (Table 1). The median follow-up after proton radiotherapy was 3.83 (2.94-4.75) years (Table 2).

**Table 1:** Baseline Characteristics between Groups

Baseline Characteristics	No Optic Neuropathy	RION	Tumor Compression	Other Cause	*P* Value∗
Number of patients	50	39	13	1	
Sex (male), n (%)	24 (48.0%)	14 (35.9%)	3 (23.1%)	0 (0.0%)	0.286
Diabetes, n (%)	4 (8.0%)	3 (7.7%)	3 (23.1%)	0 (0.0%)	1
Hypertension, n (%)	11 (22.0%)	15 (38.5%)	5 (38.5%)	0 (0.0%)	0.105
Smoking, n (%)	13 (27.1%)	14 (36.8%)	1 (7.7%)	0 (0.0%)	0.359
Type of tumor, CNS (%) (vs. head and neck)	34 (68.0%)	25 (64.1%)	12 (92.3%)	1 (100.0%)	0.822
BCVA, ETDRS, median (IQR)	85.00 (79.00–90.00)	83.00 (79.00–88.50)	79.00 (76.00–85.00)	85.00 (85.00–85.00)	0.523
VF perimetry, MD, median (IQR)	–1.54 (–3.79 to –0.59)	–1.76 (–7.11 to –0.44)	–2.15 (–3.73 to –1.00)	–0.11 (–0.11 to –0.11)	0.750
VF perimetry, PSD, median (IQR)	3.54 (2.76–6.08)	4.01 (2.65–6.49)	4.65 (3.64–7.28)	7.60 (7.60–7.60)	0.942
VEP P100 peak, latency, median ms (IQR)	108.50 (104.00–116.38)	109.50 (104.75–115.25)	108.00 (104.50–111.50)	102.50 (102.50–102.50)	0.920
VEP P100 peak, amplitude, median ms (IQR)	5.53 (3.76–7.15)	5.55 (3.92–8.41)	5.01 (4.74–6.82)	5.84 (5.84–5.84)	0.611
RNFL, median thickness (IQR)	88.89 (77.12–102.93)	79.00 (74.50–83.50)	84.00 (74.00–88.00)	72.00 (72.00–72.00)	0.604
Median follow-up, years (IQR) from initial diagnosis of tumor†	5.28 (3.84–7.51)	5.81 (3.53–10.87)	12.67 (5.18–11.17)	7.64 (7.64–7.64)	0.157
Median ophthalmological follow-up, years (IQR)	3.85 (3.11–4.80)	3.62 (2.59–4.27)	3.72 (2.84–4.80)	4.46 (4.46–4.46)	0.504

BCVA = best-corrected VA; IQR = interquartile range; MD = mean deviation; PSD = pattern standard deviation; RION = radiation-induced optic neuropathy; RNFL = retinal nerve fiber layer; VEP = visual evoked potential; VF = visual field.

∗ Wilcoxon test *P* values for continuous variable and Fisher exact test *P* values for categorical variable between the no optic neuropathy group and the RION group.

† Meningioma, pituitary adenoma, etc., can be monitored for years before radiotherapy is initiated, resulting in long median follow-up.

### Cross-Sectional Differences at Nadir

Longitudinal changes across groups are presented in Table 2. At the time of worst impairment, patients with RION exhibited significantly worse BCVA at nadir compared with patients without optic neuropathy. Values of VF MD at nadir and change in VF MD from baseline, RNFL thickness, or VEP parameters did not show marked differences (Table 2).

**Table 2:** Ophthalmologic Parameters Following Proton Therapy in Patients without RION and with RION

	No Optic Neuropathy (n = 50)	RION (n = 39)
Absolute Values at Nadir		
Median BCVA at nadir (letters)	85 (79.25–88.75)	79 (74.5–83.5)
VF MD at nadir (dB)	–2.04 (–3.55 to –0.94)	–2.47 (–11.83 to –1.04)
VF PSD at MD nadir (dB)	3.41 (2.69–5.31)	3.29 (2.66–6.27)
VEP amplitude at nadir (microvolts)	4.55 (3.22–5.54)	4.48 (2.47–7.38)
RNFL thickness at nadir (microm)	88.40 (70.05–98.46)	85.51 (72.75–98.23)
Longitudinal changes from baseline to nadir		
Change in BCVA at nadir (letters)	–3.5 (–8 to 0)	–6 (–8.5 to –2.5)
Change in VF MD at nadir (dB)	–0.66 (–2.02 to 0.13)	–1.24 (–2.59 to –0.20)
Change in VF PSD at MD nadir (dB)	3.41 (2.69–5.31)	3.44 (2.68–6.48)
Change in VEP latency at nadir (ms)	–0.12 (–0.97 to –0.57)∗	–0.09 (–1.38 to 0.92)‡
Change in VEP amplitude at nadir (microvolts)	–1.72 (–3.22 to –0.46)	–1.35 (–3.80 to –0.19)
Change in RNFL thickness at nadir (microm)	–3.15 (–5.21 to –1.43)	–4.19 (–9.08 to –2.09)

BCVA = best-corrected VA; MD = mean deviation; microm = micrometers; PSD = pattern standard deviation; RION = radiation-induced optic neuropathy; RNFL = retinal nerve fiber layer; VEP = visual evoked potential; VF = visual field.

Data are presented as median (Q1–Q3). Absolute values are shown in the upper section, whereas longitudinal changes represent the change from baseline to nadir for each patient.

∗ Wilcoxon rank-sum test comparing the no optic neuropathy and RION groups.

### Performance of Multimodal and Single-Modality Assessment

**Figure 2:**
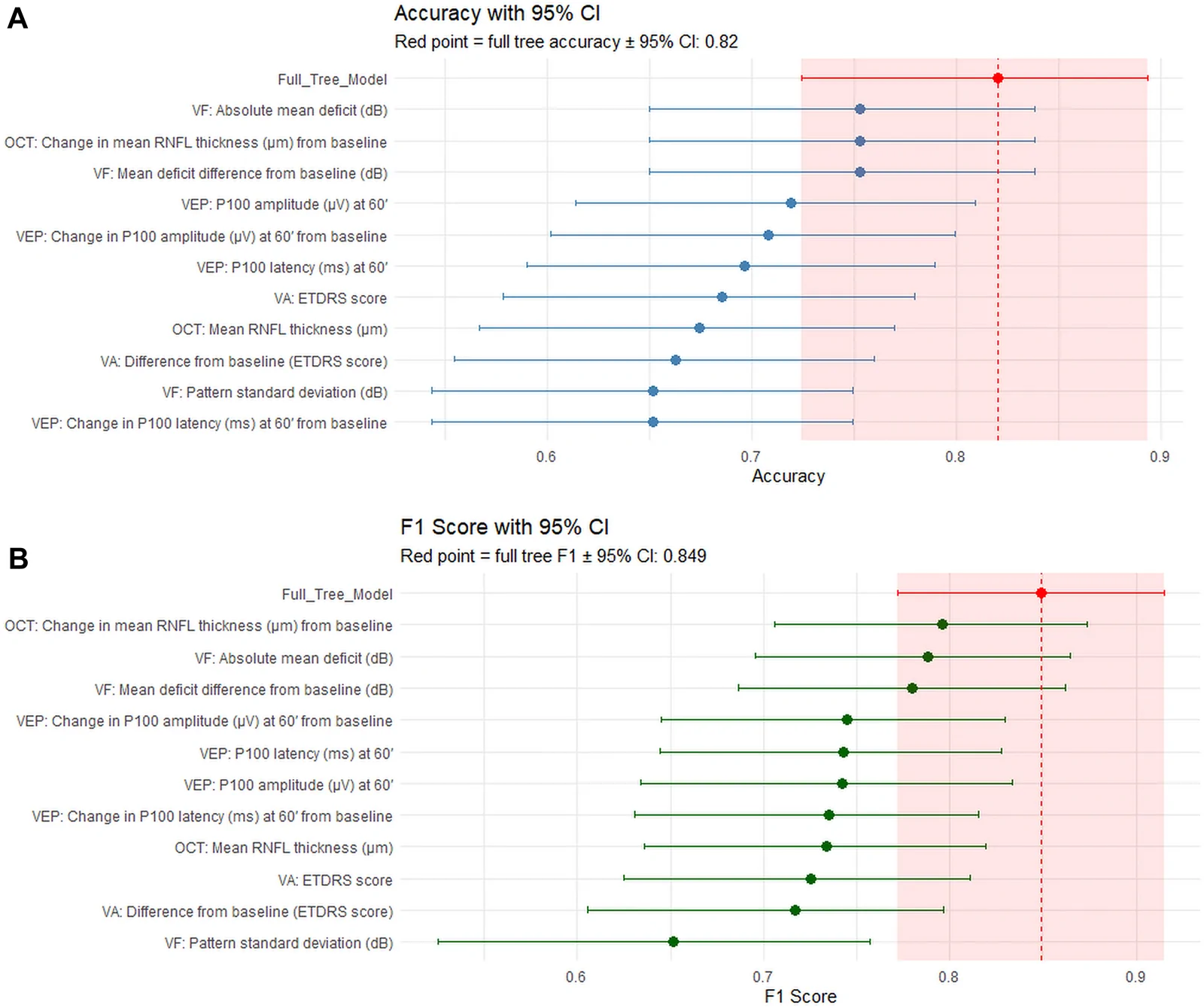
Comparison of accuracy **(A)** and F1 scores **(B)** for single-variable decision tree models versus the full multivariable decision tree model for predicting RION. Each blue/green point represents the accuracy/F1 score of a single predictor variable, with error bars indicating the uncertainty interval. The red points and error bars correspond to the full tree model's F1 score and its 95% CI. CI = confidence interval; RION = radiation-induced optic neuropathy; RNFL = retinal nerve fiber layer; VF = visual field; VA = visual acuity; VEP = visual evoked potential.

Performance of multimodal versus single-modality assessment, reported as the primary metric, was modest for the all-variable model (mean area under the curve 0.64 across folds; Table S1, available at www.ophthalmologyscience.org), consistent with some overfitting in a small cohort. When considered individually, VF parameters (MD and PSD) showed the strongest single-variable performance for RION identification (accuracy 75%–79%; F1 score 0.78–0.81). Visual acuity demonstrated moderate discriminative value; RNFL thickness and VEP parameters provided more limited standalone performance. Combining all modalities yielded higher apparent-sample performance (accuracy 81.7% [95% CI: 73.9–88.1]; F1 score 0.83 [95% CI: 0.75–0.89]; Fig 2), but this did not translate into clearly superior cross-validated discrimination compared with VF parameters alone. In-sample estimates should therefore be interpreted as secondary and exploratory.

### Relative Clinical Contribution of Ophthalmologic Parameters

**Figure 3:**
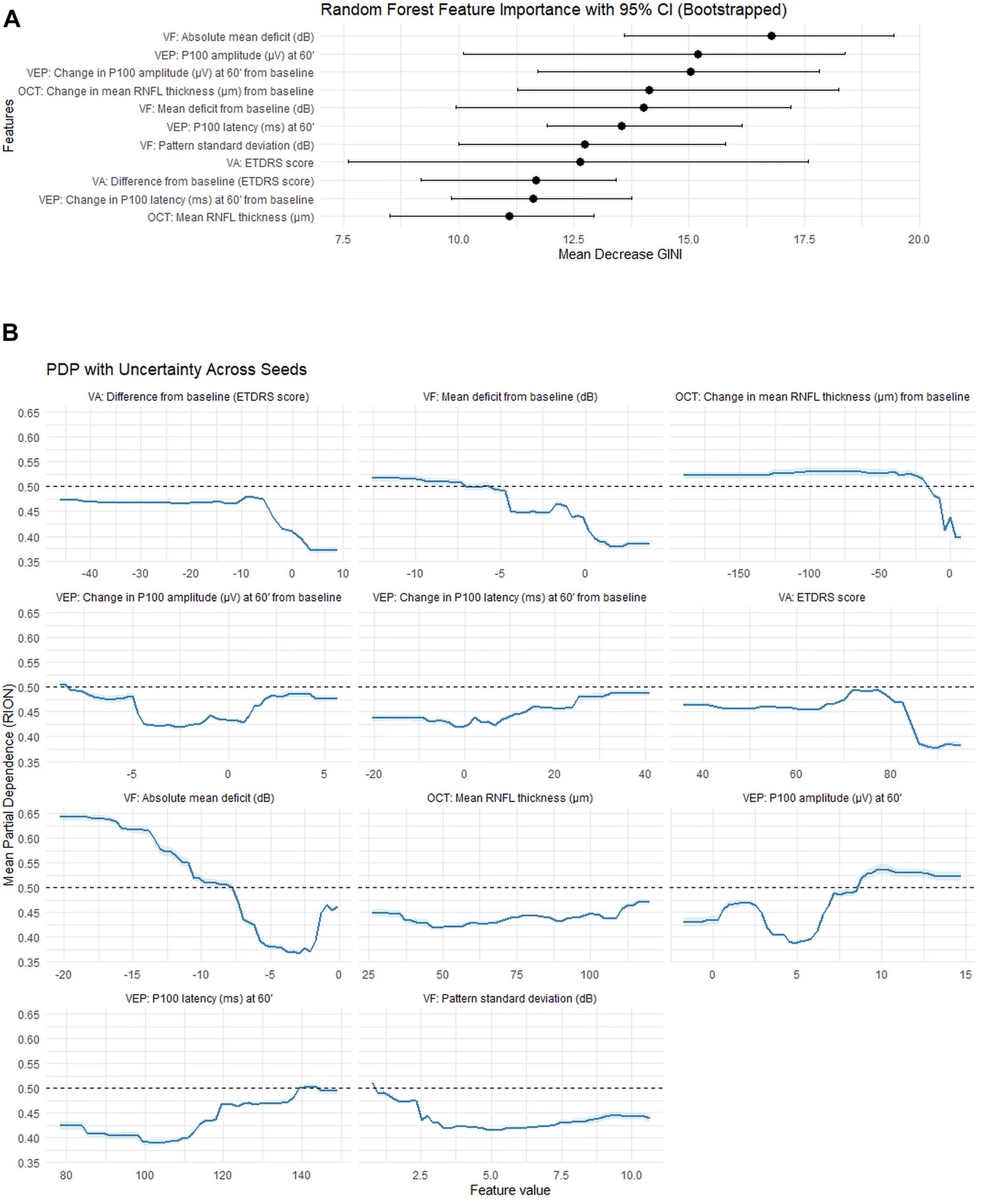
Mean feature importance **(A)** and PDPs **(B)** across 20 random forest models. Points indicate the average importance of each variable, and error bars represent ± standard deviation across models. The solid blue line represents the mean predicted probability across multiple random forest models. The y-axis shows the mean predicted probability of belonging to an RION for a given value of the feature (x-axis), averaged over all other features in the model: 0.5 represents that the model is uncertain (equal chance of an RION or no RION); >0.5 represents that the model leans toward predicting an RION; and <0.5 represents that the model leans toward predicting no RION. The blue shaded ribbon shows the ±1 standard deviation range across bootstrap seeds, indicating uncertainty in the partial dependence estimate. CI = confidence interval; PDP = partial dependence plot; RION = radiation-induced optic neuropathy; RNFL = retinal nerve fiber layer; VA = visual acuity; VEP = visual evoked potential; VF = visual field.

Visual field MD emerged as the most influential parameter (importance score: 16.8 [95% CI: 13.6–19.4]) followed by electrophysiological measures VEP P100 amplitude (15.2 [95% CI: 10.1–18.4]) and latency (95% CI: 13.5 [11.9–16.2]), VF PSD (95% CI: 12.7, [10.0–15.8]), VA (95% CI: 12.6 [7.60–17.6]), and RNFL thickness (11.1 [95% CI: 8.49–12.9]). Longitudinal changes in these parameters (Table 3) were among the most important features identified by the random forest models, suggesting that dynamic assessments may provide complementary information to static measurements: change in RNFL thickness (14.1 [95% CI: 11.3–18.3]), change in VF MD (14.0 [95% CI: 9.94–17.2]), change in P100 amplitude (15.0 [95% CI: 11.7–17.8]), change in VA (11.7 [95% CI: 9.18–13.4]), and change in VEP latency (11.6 [95% CI: 9.8–13.7]) (Fig 3).

**Table 3:** Summary Statistics of Patient Data Stratified by Postradiotherapy Interval and RION Status

After Radiotherapy Period	RION	n	Visual Acuity	Visual Field		Visual Evoked Potential P100 Peak		RNFL
			ETDRS	Mean Deficit	PSD	Latency	Amplitude	Thickness
0–0.5 yrs	No	18	0 (–5 to 4)	0.028 (–0.3 to 0.31)	0.029 (–0.47 to 0.58)	–2 (–4.5 to 2)	–0.52 (–1.7 to 0.92)	1.1 (–1.2 to 5.8)
0–0.5 yrs	Yes	16	2 (0–2)	–0.47 (–5.3 to 0.077)	0 (–1.4 to 0.31)	3 (1.5–9.2)	–2.3 (–4.9 to –0.66)	–1.7 (–32 to 0)
0.5–1 yrs	No	26	1 (–6 to 4)	0.15 (–3.2 to 0.64)	0.1 (–1.1 to 1.9)	1 (–2 to 6)	–0.32 (–2.1 to 0.39)	0.0024 (–2.3 to 6)
0.5–1 yrs	Yes	21	1 (–2 to 2)	–0.3 (–9.3 to 0.71)	–0.096 (–2.1 to 0.67)	0.5 (–0.75 to 9.5)	–1.4 (–6.7 to –0.51)	–2.1 (–35 to 0.16)
1–2 yrs	No	25	0 (–3 to 2)	–0.56 (–0.98 to 0.62)	0.037 (–1.3 to 1.3)	2 (0.5–4.5)	–1.1 (–2.4 to 1.9)	–1.6 (–3.9 to 0.67)
1–2 yrs	Yes	14	3 (–2 to 8)	–0.35 (–1.4 to 0.045)	–1.4 (–3.2 to 0.79)	0.5 (–6.9 to 9)	–2 (–7.2 to –0.55)	–4 (–7.9 to –0.34)
2–5 yrs	No	14	–3 (–7.5 to 4)	–1 (–3.2 to –0.44)	0.48 (–0.69 to 1.7)	3 (0.25–6.8)	0.7 (–1.3 to 2.4)	–1.7 (–8.2 to 1.1)
2–5 yrs	Yes	5	–1 (–4.8 to 2)	–0.99 (–9.9 to –0.35)	–0.085 (–3.4 to 0.72)	3 (–3 to 11)	–1.4 (–6.5 to –0.98)	–10 (–42 to –1.8)

PSD = pattern standard deviation; RION = radiation-induced optic neuropathy; RNFL = retinal nerve fiber layer.

Continuous variables are summarized as medians (Q1–Q3).

Partial dependence analyses suggested that worsening VF MD and PSD (PDP = 0.25) and declining VA (PDP = 0.24) were associated with the largest increases in RION probability, while VEP and RNFL measures contributed moderate (PDP = 0.19) but complementary information. P100 amplitude had the smallest independent effect (PDP = 0.08) (Fig S1, available at www.ophthalmologyscience.org).

### Relative Contribution of Individual Ophthalmic Assessments in Detecting RION by Time Point

Patients with RION exhibited greater impairments in VA, VF, VEP, and RNFL thickness than did those without RION, with these differences evolving over time after radiotherapy. Across time windows, functional and electrophysiological abnormalities appeared more prominent in the earlier intervals, whereas structural RNFL changes showed greater prominence at later intervals; these are group-level observations and do not establish temporal sequence at the individual patient level (Table 2, Table 3).

**Figure 4:**
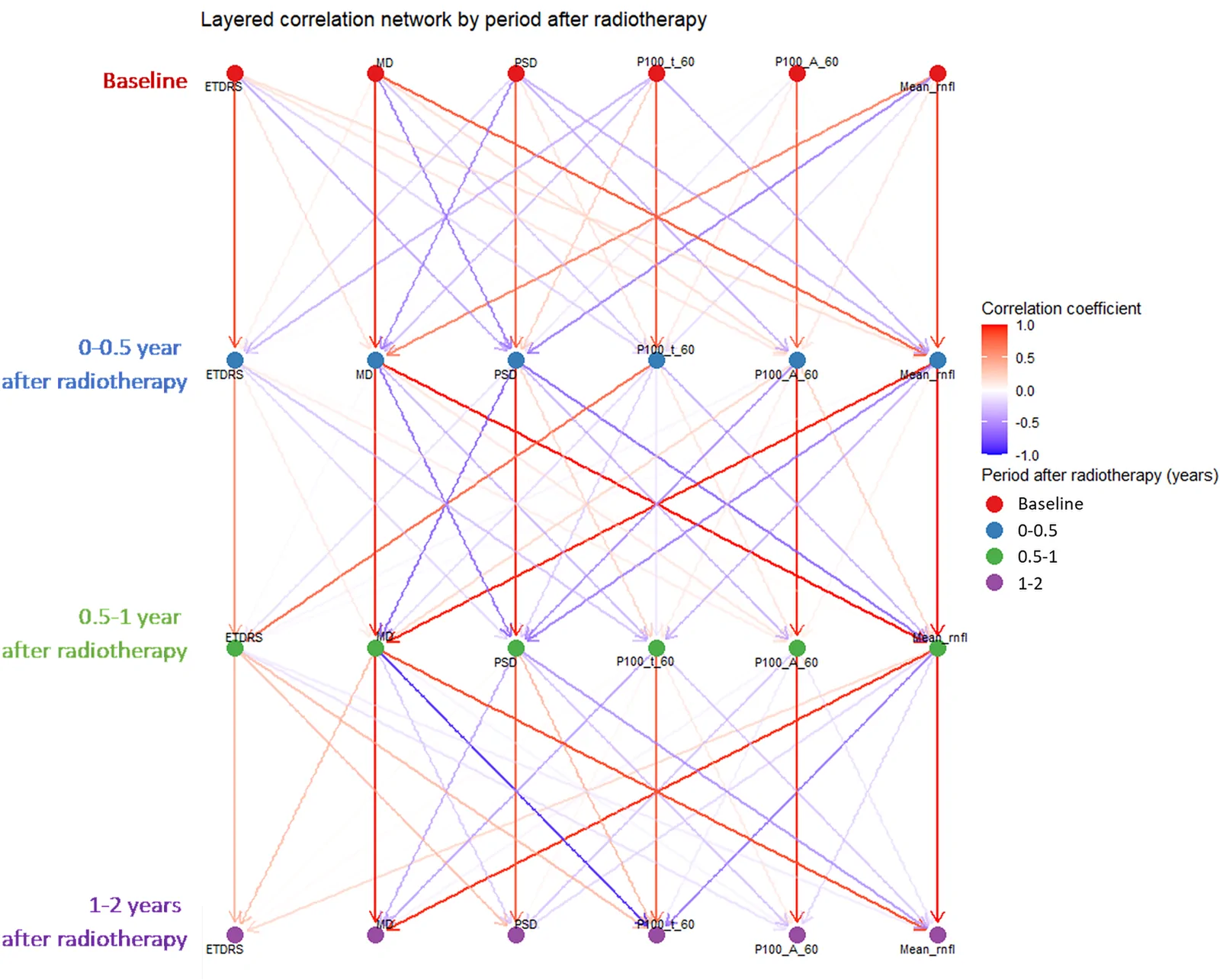
Layered correlation network by period after radiotherapy. MD = mean deviation; PSD = pattern standard deviation.

The relative importance of ophthalmologic parameters varied over time (Table 3). Within the first 6 months after radiotherapy, functional and electrophysiological changes, particularly VF deficits and VEP abnormalities, were most prominent. Between 1 and 2 years, RNFL thinning became the dominant contributor, consistent with progressive axonal degeneration. At later time points, overall discriminative ability decreased, which should be interpreted cautiously because of the limited number of patients available in later time windows (Fig 4).

Longitudinal correlation analyses demonstrated strong persistence of abnormalities within each modality over time, with selected cross-modal relationships suggesting that early functional impairment may be associated with later electrophysiological and structural changes; these correlations are exploratory and should not be interpreted as establishing causal or temporal directionality (Fig S1).

## Discussion

This prospective multimodal study provides a characterization of clinically diagnosed RION following proton radiotherapy and supports a more comprehensive approach to ophthalmologic monitoring than VA-based assessment alone. Our time-window analyses suggest that different modalities may capture injury at different stages: functional changes appear more prominent within the first year, while structural RNFL thinning is more evident in the second year. Whether this pattern reflects a true temporal sequence at the individual patient level requires prospective confirmation with individual-level event timing data. This group-level pattern is consistent with the established pathophysiology of RION, which involves radiation-induced microvascular injury, chronic hypoperfusion, glial and myelin damage, and subsequent axonal loss.^16^, ^17^, ^18^ Endothelial dysfunction and ischemia are thought to initiate conduction abnormalities along the visual pathway, while progressive demyelination and axonal degeneration then lead to RNFL thinning and ultimately VA loss. The multimodal patterns observed here are broadly consistent with this framework. The limited sensitivity of VA for capturing the full spectrum of radiation-related optic nerve injury underscores a key limitation of current CTCAE-based grading,^5^ which is anchored to VA loss and may underestimate or delay the recognition of RION.

The predominance of VF parameters as indicators of RION likely reflects their ability to capture the integrated functional consequences of optic nerve damage across spatial domains. Visual field testing is sensitive to both localized and diffuse dysfunction and may therefore reveal clinically meaningful impairment even when structural loss remains below the detection threshold of OCT. Although RNFL thinning is a hallmark of axonal degeneration, it represents a relatively late manifestation of disease and offers limited opportunity for therapeutic intervention once established. Similarly, VEP abnormalities provide sensitive markers of conduction impairment that may occur in the absence of subjective visual symptoms, contributing to their apparent dissociation from cross-sectional clinical endpoints.

The absence of marked cross-sectional differences between RION and non-RION groups for certain parameters in our cohort underscores the limitations of static, threshold-based comparisons. Rather than indicating a lack of biological relevance, this finding highlights the subclinical and progressive nature of early radiation-induced optic nerve injury and emphasizes the importance of longitudinal, multimodal assessment to capture dynamic changes over time.

Previous attempts to predict RION have primarily focused on clinical and dosimetric parameters, including maximum point doses and dose‒volume relationships for the optic nerves and chiasm. Dose–response associations were first characterized by Mayo et al^19^ with conventional radiotherapy and Köthe et al^4^ reported incremental improvement in predictive performance when dosimetric and clinical features were combined in proton therapy cohorts.^20^ However, such approaches remain limited in their ability to detect subthreshold or early injuries and do not fully account for interindividual biological susceptibility. In specific tumor populations, such as nasopharyngeal carcinoma, psychophysical testing, RNFL measurements, and electrophysiological assessments have demonstrated radiation-related changes, including delayed VEP conduction.^7^ By integrating multiple complementary ophthalmologic assessments within a prospective framework with standardized longitudinal follow-up, the present study extends this body of evidence and supports the potential added value of a multimodal approach over single-test evaluation.

Our findings have important clinical implications. First, they challenge the adequacy of the current CTCAE-based grading systems, which rely heavily on VA only and may substantially delay the recognition of RION. If confirmed prospectively, earlier identification could create a therapeutic window for interventions that are unlikely to be effective once axonal loss is established.^2^^,^^21^^,^^22^ Third, systemic multimodal monitoring could improve the accuracy of toxicity reporting, refine normal tissue complication probability models, and inform adaptive radiotherapy strategies in high-risk patients.

This study also provides a framework for integrating ophthalmologic biomarkers into survivorship care and future clinical trials. By characterizing the relative prominence of functional, electrophysiological, and structural changes across postradiotherapy time windows, our approach may facilitate more precise localization of radiation-induced injury and support emerging voxel-based or functional subunit-based planning strategies.^23^

Several limitations should be acknowledged. First, RION diagnosis lacks a universally accepted gold standard and therefore relied on clinician assessment in this study. While clinically relevant, this assessment may introduce subjectivity. Secondly, the resource-intensive nature of multimodal testing may not be available in all centers. Thirdly, the study was restricted to proton therapy settings, and extrapolation to photon therapy populations should be made with caution. Nevertheless, the underlying pathophysiology of RION is not modality-specific, supporting the broader relevance of our findings.

In conclusion, this prospective multimodal study demonstrates that VF is the strongest single modality for identifying RION after proton radiotherapy and that combining modalities provides higher apparent-sample discrimination than VA alone. The cross-validated performance indicates that these findings are hypothesis-generating; larger, prospective studies are required before multimodal monitoring can be recommended for routine clinical implementation. Visual field MD represents a promising candidate marker for future studies.

## Data Availability

Data are available upon reasonable request.
