# Supplementary material for: Multimodal Characterization of Radiation-Induced Optic Neuropathy after Proton Radiotherapy
Source: Ophthalmol Sci. 2026 Aug 10;6(10):101359. doi: 10.1016/j.xops.2026.101359 (PMC13583721; doi:10.1016/j.xops.2026.101359)
Supplement: Supplementary file 1 [file mmc1.pdf]

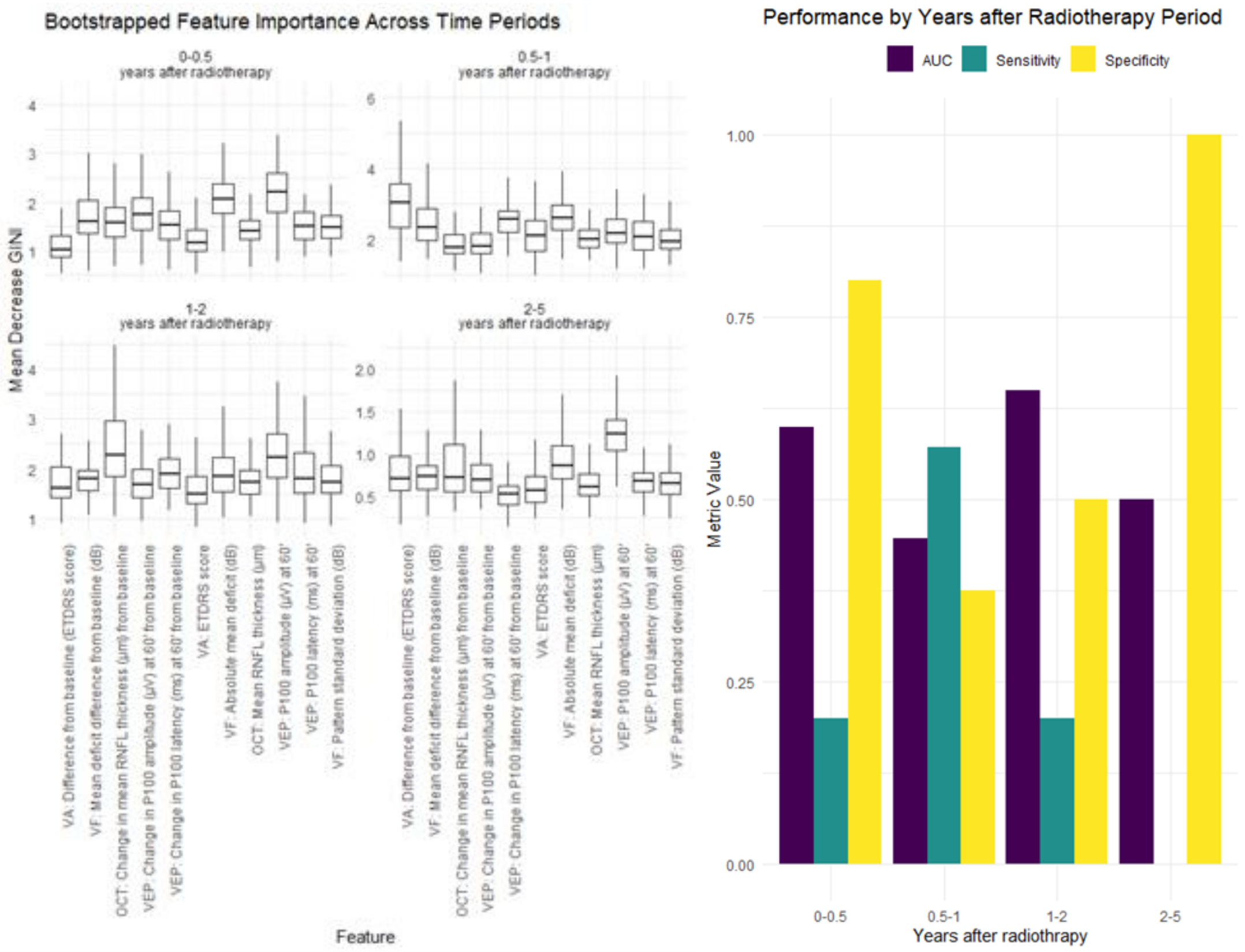

Figure S1. Bootstrapped feature importance measured by the mean decrease in Gini impurity (A) and model performance (B) across different postradiotherapy intervals.

Table S1. Five-fold cross-validation performance of predictive models

Variable	Mean AUC	Min AUC	Max AUC	Mean Brier	Min Brier	Max Brier
**Median BCVA at nadir**	0.62	0.53	0.81	0.26	0.18	0.34
**Change in BCVA at nadir vs baseline**	0.71	0.61	0.85	0.28	0.20	0.47
**VF MD at nadir**	0.64	0.48	0.75	0.23	0.20	0.27
**Change in VF MD at nadir**	0.68	0.56	0.78	0.25	0.19	0.40
**VF PSD at MD nadir**	0.48	0.36	0.54	0.27	0.24	0.30
**VEP latency at nadir**	0.59	0.53	0.69	0.27	0.22	0.32
**Change in VEP latency at nadir**	0.49	0.47	0.50	0.25	0.25	0.28
**VEP amplitude at nadir**	0.61	0.53	0.73	0.25	0.21	0.29
**Change in VEP amplitude at nadir**	0.54	0.40	0.73	0.29	0.23	0.37
**RNFL thickness at nadir**	0.58	0.43	0.81	0.30	0.25	0.40
**Change in RNFL thickness at nadir**	0.61	0.51	0.69	0.28	0.24	0.36
**All variables**	**0.64**	**0.54**	**0.78**	**0.25**	**0.17**	**0.34**
